# Melanom in Assoziation mit einem Nävus oder „naevus sur naevus“?

**DOI:** 10.1007/s00105-025-05534-9

**Published:** 2025-07-10

**Authors:** Julia Resch, Isabella Fried, Rainer Hofmann-Wellenhof

**Affiliations:** Universitätsklinik für Dermatologie und Venerologie, Medizinische Universität Graz, Auenbruggerplatz 8, 8036 Graz, Österreich

## Anamnese

Eine 19-jährige Patientin wird im Rahmen einer Hautkrebsvorsorgeuntersuchung vorstellig. Sie hat multiple melanozytäre, teils gruppierte Nävi und lässt diese seit 4 Jahren in regelmäßigen Abständen kontrollieren.

Anamnestisch gibt sie an, dass ihr ein ungewöhnlicher melanozytärer Nävus am Rücken aufgefallen sei. Sie habe sich an dieser Stelle vor 3 Monaten verletzt, danach kam es zur Veränderung. Weitere Auffälligkeiten bestanden zum Zeitpunkt der Vorstellung nicht.

## Hautbefund

**Abb. 1:**
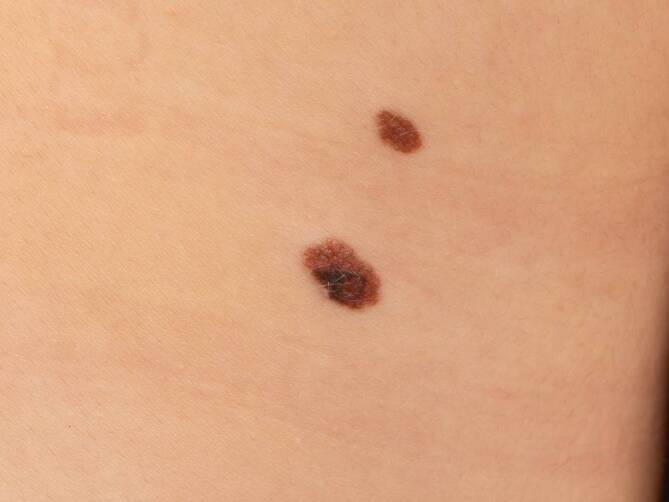
Klinisches Bild der melanozytären Läsion

Neben mehreren unauffälligen melanozytären Nävi am Rücken zeigte sich infraskapulär rechts eine gering unregelmäßig begrenzte, ovale mittelbraune, flache Plaque, die im Randbereich eine unscharf begrenzte, dunkelbraune unregelmäßige Pigmentierung zeigte (Abb. 1).

**Abb. 2:**
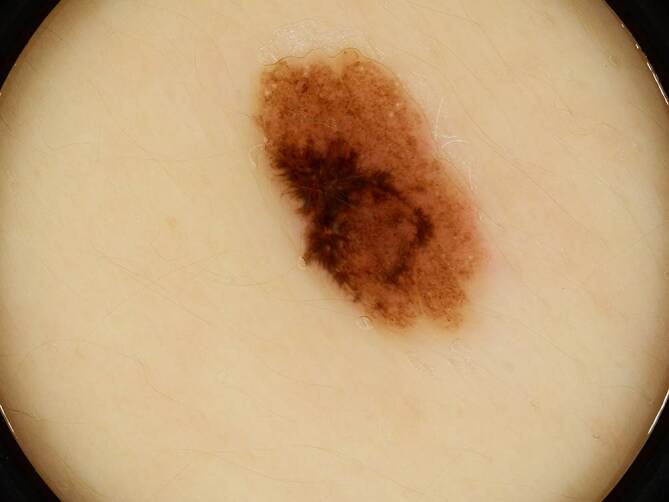
Dermatoskopisches Bild: im lateralen Bereich asymmetrische schwarze Pigmentierung mit „starburst pattern“ und radialen Streifen zur Peripherie hin. Homogene Pigmentierung mit dunkelbraunen regelmäßigen Schollen im hellen Teil der melanozytären Läsion

Im Dermatoskop war im lateralen Bereich eine asymmetrisch schwarze Pigmentierung mit „starburst pattern“ und radialen Streifen zur Peripherie hin sichtbar. Der helle Teil der melanozytären Läsion wies eine homogene Pigmentierung mit dunkelbraunen regelmäßigen Schollen auf (Abb. 2).

## Verlauf

Anhand dieser Befunde konnte ein Melanom in Assoziation mit einem melanozytären Nävus nicht ausgeschlossen werden. Daher erfolgte eine Totalexzision.

## Diagnose

Die Histologie ergab einen atypischen melanozytären Nävus vom Compound-Typ mit teils kongenitalem Wachstumsmuster. Die Läsion wies fokal Merkmale eines Rezidivnävus mit Fibrosierungszonen auf.

In Zusammenschau der Befunde wurde ein Rezidivnävus im melanozytären Nävus, ein sog. „naevus sur naevus“, als Diagnose gestellt.

## Diskussion

Rezidivnävi sind gutartige melanozytäre Neubildungen ohne nachweislich erhöhtes Risiko für eine maligne Entartung [1]. Es kommt zu einem neuerlichen Wachstum von Melanozyten nach totaler oder subtotaler Entfernung mittels Laserbehandlungen, Shaving, Stanzbiopsien oder Verletzungen, die zu Narben führen. Die histologischen Merkmale können jenen eines regressiven oder Rezidivmelanoms ähneln [2–4]. Häufiger sind weibliche Patienten betroffen [2].

In der Diagnostik ist es entscheidend festzustellen, ob die Pigmentierung infolge einer unvollständigen Exzision des melanozytären Nävus oder durch ein Nävus-assoziiertes Melanom auftritt.

Rezidivierende melanozytäre Nävi sind häufig am Rücken lokalisiert, treten typischerweise innerhalb von 6 Monaten nach dem Trauma auf, und die Betroffenen sind jünger [4–6]. In der retrospektiven Beobachtungsstudie von Blum et al. zeigte sich, dass Rezidivmelanome häufiger bei PatientInnen über 30 Jahren auftreten und hauptsächlich an Kopf und Hals lokalisiert sind [4]. Letzteres stimmt mit den Studienergebnissen von Heck et al. überein [3].

Als stärkstes Anzeichen für ein rezidivierendes Melanom gilt eine Repigmentierung, die den Narbenrand überschreitet, bei rezidivierenden melanozytären Nävi erfolgt die erneute Pigmentierung innerhalb der Narbe [1, 4].

Im beschriebenen Fall könnte anhand der anamnestischen Angaben (Patientenalter, zeitliche Entwicklung) bereits eine klinische Verdachtsdiagnose gestellt werden. Dermatoskopisch zeigten sich jedoch Melanom-typische Merkmale. Es wurde daher differenzialdiagnostisch auch an ein Melanom in Assoziation mit einem kongenitalen Nävus gedacht und die Läsion entfernt.

Zusammenfassend lässt sich sagen, dass in Fällen, bei denen klinisch und dermatologisch ein Melanom nicht mit Sicherheit ausgeschlossen werden kann, eine Exzision erfolgen sollte.

Außerdem ist es für die histologische Diagnostik wichtig, anamnestische Daten miteinfließen zu lassen.
